# Influence of nutrition claims on different models of front-of-package nutritional labeling in supposedly healthy foods: Impact on the understanding of nutritional information, healthfulness perception, and purchase intention of Brazilian consumers

**DOI:** 10.3389/fnut.2022.921065

**Published:** 2022-09-23

**Authors:** Sarah Morais Senna Prates, Ilka Afonso Reis, Carlos Felipe Urquizar Rojas, Carla Galvão Spinillo, Lucilene Rezende Anastácio

**Affiliations:** ^1^Food Science Post-Graduation Program, Department of Food Science, Universidade Federal de Minas Gerais, Belo Horizonte, Brazil; ^2^Department of Statistics, Institute of Exact Sciences, Universidade Federal de Minas Gerais, Belo Horizonte, Brazil; ^3^Laboratory of Information System Design, Design Post-Graduation Program, Department of Design, Universidade Federal do Paraná, Curitiba, Brazil

**Keywords:** food labeling, nutrition policy, FOP, warning labels, consumer research, consumer perception, health halo, positivity bias

## Abstract

Nutrition claims are positive information about foods, which are widely used as a marketing strategy on labels. On the contrary, front-of-package nutritional labeling (FoPNL) aims to make it easier for consumers to understand the nutritional composition of foods and favor healthy food choices. However, the concomitant presence of nutrition claims and FoPNL may hinder the understanding, judgment, and choices of consumers at the moment of purchase. Therefore, the objective of this study was to evaluate the influence of nutrition claims on the efficacy of FoPNL models in the understanding of nutritional information, healthfulness perception, and purchase intention of Brazilian consumers. It was an experimental cross-sectional study carried out using an online questionnaire, with a total of 720 participants randomly divided into four FoPNL conditions: control, octagon, triangle, and magnifying glass. Each participant looked at 12 food packages, which were produced following the factorial design: (i) food category (cereal bar, whole grain cookies, and snacks); (ii) product type (containing one critical nutrient × containing two critical nutrients); and (iii) nutrition claims (present × absent). The comprehension of nutritional information was evaluated through the identification of excessive nutrients, and the healthfulness perception and purchase intention were evaluated using a seven-point scale. The results indicated that the presence of FoPNL increased the understanding of the information and reduced healthfulness perception and purchase intention. The presence of nutrition claims influenced the three outcomes, decreasing the probability of understanding information about food composition by 32% (OR 0.68, 95% confidence interval 0.58–0.78, *p* < 0.01) and significantly increasing (*p* < 0.05) average health scores (1.95–2.02) and purchase intention (2.00–2.05). Nonetheless, the interaction “FoPNL × claims” was not significant, which indicated that claims act independently. All FoPNL models were more effective than the control. For the least healthful type of product (two nutrients in excess), the octagon and triangle models were superior to the magnifying glass, regarding the outcome of healthfulness perception. The results prove the efficacy of FoPNL in consumer understanding and judgment. Despite the positive effects of FoPNL, it did not cancel the positivity bias generated by the claims.

## Introduction

In general, nutrient declarations, especially those related to excessive nutrients considered critical (such as added sugars, saturated fat, and sodium), are hard to find and interpret and are therefore rarely used by consumers at the moment of purchase (1–3). For this reason, the front-of-package nutritional labeling (FoPNL) was proposed to make it easier for consumers to understand the nutritional information of foods (4, 5). Besides improving the comprehension of nutritional information, FoPNL encourages healthier food choices as well as product reformulations by the industry toward less nutritionally unbalanced options (6). Thus, it may aid in the reduction of obesity risk and non-communicable diseases (7).

As recommended by international health bodies (8–10), FoPNL has been implemented in different countries with differences in design, type of information provided, and the degree/level of guidance provided for consumers to communicate the nutritional content and relative healthfulness of the foods (11, 12). Among the different models of FoPNL, nutritional warning models, which provide direct information about the excessive content of harmful nutrients, using text-based seals (13), have performed better than other FoPNL models (11, 13–16). This happens because nutritional warning models better serve the purpose of FoPNL: to allow consumers to correctly, quickly, and easily identify products that contain excessive amounts of critical nutrients, promoting more informed choices (13).

Several Latin American countries already have mandatory frontal nutritional labeling with warning models (17). In Chile (18), Mexico (19), Peru (20), and Uruguay (21), for example, the black octagon is applied to the front panel of packages to inform consumers when a product contains excessive amounts of critical nutrients (13). In Brazil, the triangle model was proposed to the regulatory body as an option for FoPNL, by design and nutrition experts (22). However, the magnifying glass was the chosen model, which will be implemented on a mandatory basis in October 2022 (23, 24). Although the magnifying glass models can promote the identification of “high in” food products as other warning models, its graphic symbol does not refer to the idea of alert or risks like other symbols, such as the octagon and the triangle, but the idea of “magnifying information, making its visualization easier, and suggesting the search for and evaluation of other information” (25).

A series of factors influence the performance and efficacy of FoPNL models, both regarding design characteristics such as color, size, and position in the package (26), and personal characteristics of consumers, such as gender, age, socioeconomic status, and interest in health (12). For instance, studies have shown that individuals with greater interest in health and healthy eating tend to check the nutritional information available on labels more frequently to make their food choices (27–30). Nutrient declarations are not the only source of information that compete for the attention of consumers who read labels to choose foods (31–33). Nutrition claims, though regulated, are used by the food industry to attract attention, promote health associations/perceptions among consumers, and increase sales (31, 32, 34, 35). According to the taxonomy developed by the International Network for Food and Obesity/Non-communicable Diseases Research, Monitoring, and Action Support (INFORMAS), food claims are classified into three categories: nutritional, health, and other claims that include, for example, claims related to the environment, such as “organic” (36).

Some studies have shown that the presence of claims on foods positively influences consumer perceptions of products (32, 37–43). However, in the literature, results concerning the effects of claims on consumer perceptions and behavior are inconsistent (44, 45). A review on the topic, which evaluated different types of claims (nutrition, health, and risk reduction), concluded that specific consumer characteristics, such as nutrition knowledge and health motivation, as well as product characteristics such as perceived healthiness, are the factors responsible for this incongruity of results (45). Furthermore, the influences of these characteristics may be limited to the food categories tested in the studies, which makes it difficult to generalize the results (45).

This positive influence generated by the presence of the claims is defined as a positivity bias or a health halo effect (44). The positivity bias occurs when the presence of information leads to better evaluations of the products by the consumer (46). The halo effect occurs when the consumer uses more specific information, for example, claims, to evaluate the product instead of other available information (46). These positive effects can generate misjudgments about the healthiness of foods (47), especially nutritionally unbalanced foods (45).

For this reason, some studies have evaluated the effects of the concomitant presence of FoPNL with other resources from the label, such as nutrition claims (32, 39, 40, 48, 49). The results of the studies are contradictory; while some have observed that FoPNL can nullify the positivity bias generated by claims (32, 41, 50), others have observed that the positive effect of claims on consumer perceptions occurs even in the presence of FoPNL (39, 40), and claims concerning the nutrients targeted by FoPNL may hinder the effectiveness of the FoPNL itself (49).

In this type of experimental design, there is still a lack of research performed on the Brazilian population (32) and, so far, there have been no studies in the literature that evaluate the influence of nutrition claims on the magnifying glass model, which will be implemented in the country (23). Therefore, the objective of this study was to evaluate the influence of nutrition claims on the efficacy of octagon and triangle FoPNL models, as determined by the Chilean legislation, and of the magnifying glass model, according to Brazilian legislation, in the understanding of nutritional information, healthfulness perception, and purchase intention of Brazilian consumers.

Given the positivity bias generated by nutritional claims on consumer perceptions (15, 32, 39, 41), and the trend toward the better performance of warning labels compared to the magnifying glass model (12, 51, 52), reported in the literature, the following hypotheses were created: (i) The presence of claims will lead to a positivity bias (greater perceptions of healthiness and purchase intent), regardless of FoPNL; (ii) the positivity bias generated by the claims will be weaker in the presence of warning labels (octagon and triangle), intermediate in the presence of the magnifying glass, and higher in the control.

## Materials and methods

### Participants

The study was performed with 720 participants who were over 18 years of age, representing the Brazilian population regarding gender, geographical region, socioeconomic status, and education characteristics, using quotas defined according to the Brazilian Institute of Geography and Statistics (53). The inclusion criteria of the study were being over 18 years of age and answering the questionnaire with the use of a computer. This last criterion excluded the participation using cellphones and tablets to ensure label visualization was as close as possible to real-sized products. The exclusion criteria were working in the food and/or nutrition field. The participants were recruited from a panel of respondents obtained from a market research company. All participants consented to participate in the research through a free and informed consent form. The study was carried out between December 2021 and February 2022, as approved by the Research Ethics Committee of the Universidade Federal de Minas Gerais (Brazil Platform—CAAE 2395020.1.0000.5149).

### Experimental design

**FIGURE 1:**
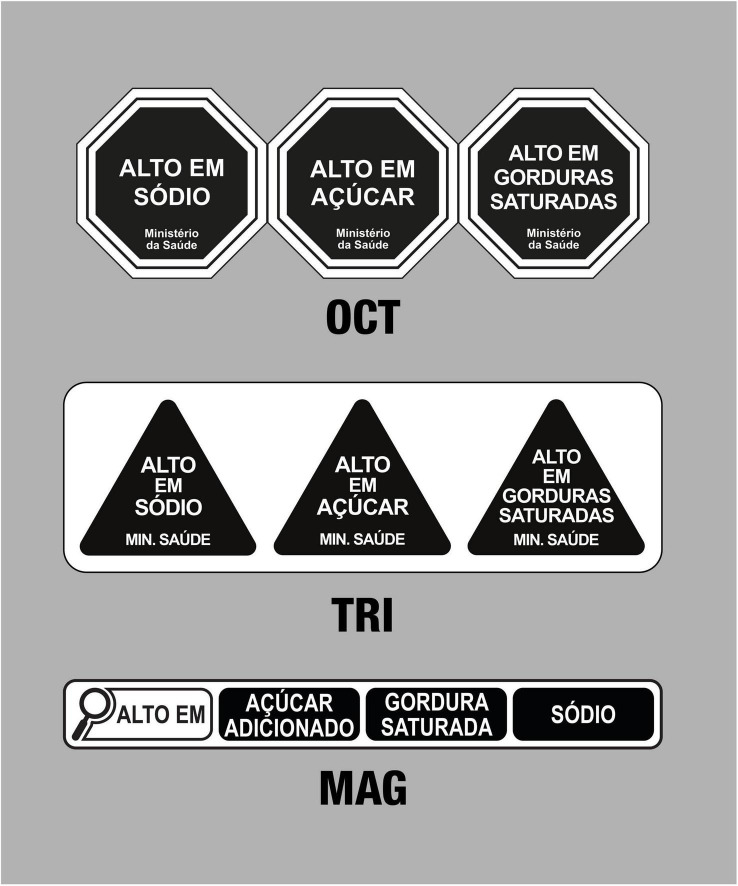
Models of front-of-package nutritional labeling tested in the study. OCT, octagon; TRI, triangle; MAG, magnifying glass.

This was an experimental, cross-sectional study, carried out through an online questionnaire, to evaluate the influence of nutrition claims on different FoPNL models. It was based on the studies by Deliza et al. (12) and Bandeira et al. (51), who evaluated the performance of different FoPNL models according to observations from Brazilian consumers, among which was a preliminary model of the magnifying glass model. The study was also based on the research by Nobrega et al. (32), who evaluated the effect of the presence of nutrition claims and warnings on the healthfulness perception of Brazilian consumers. In this study, the FoPNL models tested were black octagon, black magnifying glass, and black triangle (Figure 1). Data on three outcomes were collected: the understanding of nutritional information (correct or incorrect), healthfulness perception, and purchase intention (both measured using a seven-point scale).

**FIGURE 2:**
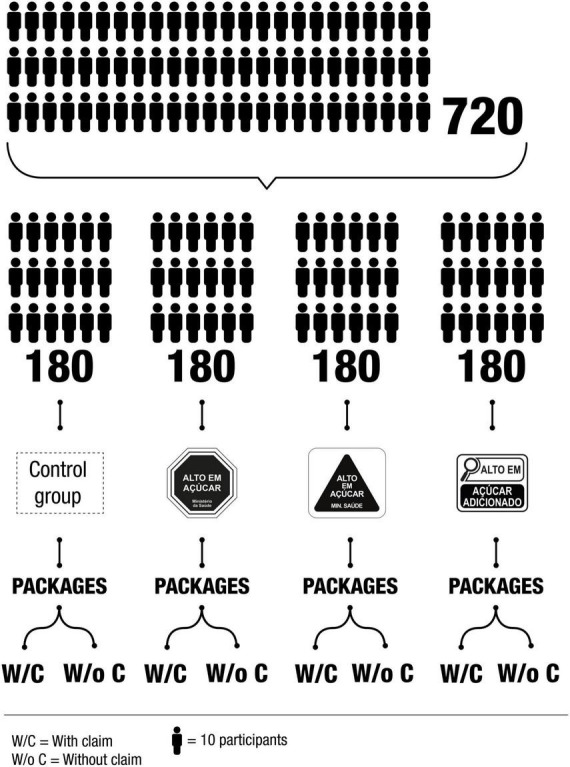
Allocation of participants in the intervention conditions of the study.

The participants were randomized in one of the four experimental conditions: control (without FoPNL), octagon, magnifying glass, and triangle (Figure 2). For each condition, the combinations between the following three factors tested in the food labels were assessed: (i) product category (cereal bar, whole grain cookies, and snacks); (ii) product type (containing one critical nutrient × containing two critical nutrients); and (iii) nutrition claims (present × absent). The combination of these three factors (3 × 2 × 2) resulted in 12 front panels of food labels, by experimental condition, and the presentation of the images varied among the participants, following a Latin square experimental design (54). Considering operational issues regarding data collection that would make it impossible to obtain preliminary data to calculate the sample size, the calculations were done assuming a continuous response to be analyzed by an ANOVA applied to the data of an experiment designed with blocks (participants) that evaluated 12 treatments (repeated measures). The number of participants in each one of the four experiments was limited to 180, resulting in a total of 720 participants (Figure 2). Since each Latin square required a minimum of 12 participants, it was replicated 180/12 = 15 times. Considering a probability of 5% of Type I Error and a test power of 90%, the number of 180 participants would be able to detect a size effect equal to or higher than 0.345 (a moderate one). All participants looked at the same food labels, with changes only in the presence/model of FoPNL.

### Stimuli

**FIGURE 3:**
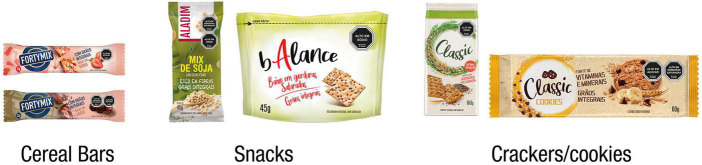
Food categories considered in the study with an example of the octagon symbol.

**FIGURE 4:**
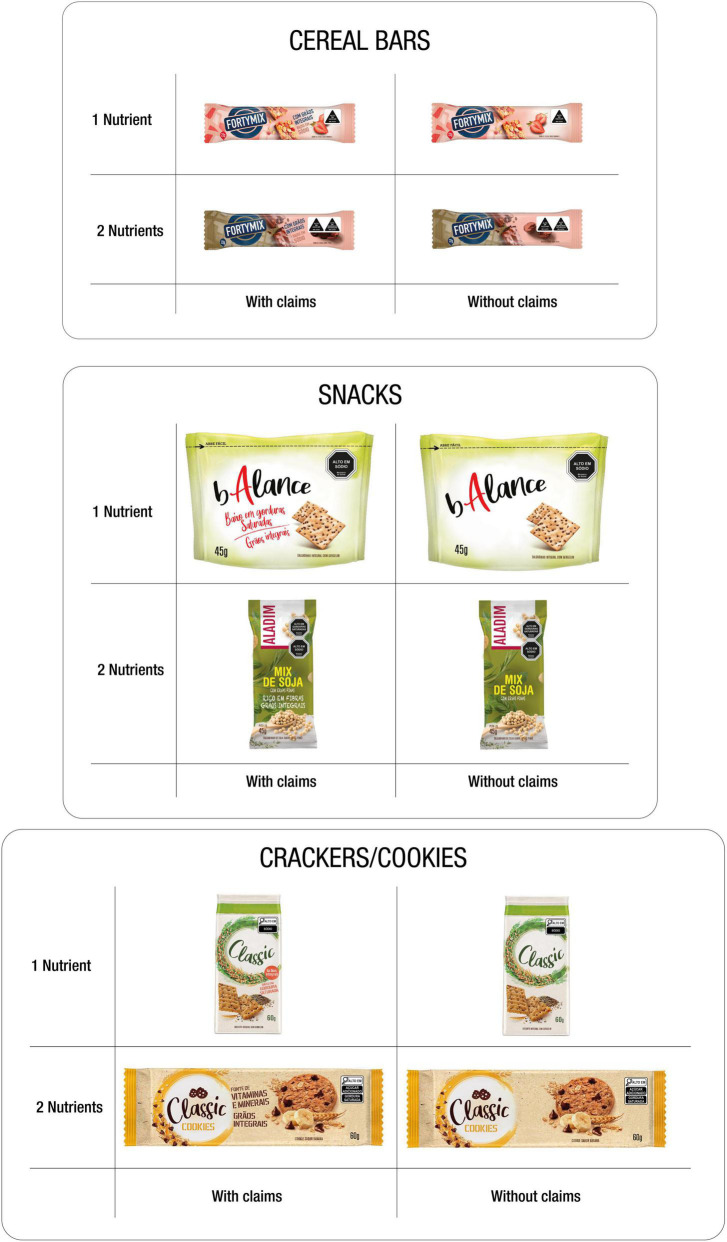
Combination of factors considered for label development: food category (cereal bars, snacks, and crackers/cookies); type of product (containing 1 or 2 nutrients in excess); nutrition claim (present vs. absent).

The three food categories (cereal bars, whole grain cookies, and snacks) with a “healthy snack” visual appeal (Figure 3) were selected due to the potential of FoPNL to modify consumer healthfulness perception and purchase intention, especially for foods that are mistakenly regarded as healthy (55), such as cereal bars, breakfast cereals, among others, which traditionally use the healthfulness “appeal” as a marketing strategy (33, 56–58). For each category, two types of products with different nutritional compositions were selected, one with only one excessive critical nutrient and another with two excessive critical nutrients (Figure 4). The presence and absence of positive nutrition claims on food labels were controlled. Two nutrition claims were included in products that were supposed to carry such information: nutrient content claim and another nutrient claim related to health, according to the INFORMAS taxonomy (36) (Figure 4). The combination of the three mentioned factors, (i) product category, (ii) product type, and (iii) nutrition claims, resulted in 12 (3*2*2) package front panels with different food labels, for each experimental condition. Table 1 shows the nutritional composition of the products used as a reference to elaborate the labels, with the respective information on FoPNL inclusion and nutrition claims.

**TABLE 1:** Nutritional composition of the products used as a reference for the study and their respective FoPNL and nutrition claim information.

Category	Type of product	Serving (g)	Nutrient content per serving					FoPNL			Nutrition claims
					
			Calories (kcal)	Total sugars (g)	Added sugars (g)*	Saturated fat (g)	Sodium (mg)	Added sugars	Saturated fat	Sodium	Nutrient claim related to health/Nutrient content claim
Cereal bar	1 nutrient	22	87	4.8	3.6	1.3	17	✓			With whole grains/Low in sodium
	2 nutrients	20	86	5.7	5.7	2.5	28	✓	✓		With whole grains/Low in sodium
Whole grain cookie	1 nutrient	21	88	2.4	2.4	0.4	130			✓	Whole grains/Low in saturated fat
	2 nutrients	20	92	6.4	4.8	1.7	40	✓	✓		Whole grains/Source of vitamins and minerals
Snack	1 nutrient	45	184	2.8	2.8	0.7	182			✓	Whole grains/Low in saturated fat
	2 nutrients	40	188	–	–	4.0	500		✓	✓	Whole grains/High in fiber

*Estimated according to the PAHO Nutrient Profile Model.

The front panels of packages with labels were developed by an information designer, using Photoshop and Illustrator software (Adobe©), to combine graphic elements (colors, typography, photography, and illustrations) from different products that exist in the Brazilian market, fictitious brands, and at least one excessive critical nutrient (added sugars, saturated fat, and sodium). Although the Chilean legislation (18) encompasses a greater number of critical nutrients in FoPNL, only the three nutrients mentioned were considered, once that is how the Brazilian legislation (23) defines it. The added sugar, saturated fat, and sodium limits considered for the inclusion/declaration in the FoPNL were also defined according to the parameters of the Brazilian legislation (23), considering the nutritional composition of the used products as a reference. Once the total and added sugars were not available in the nutritional information of the products, an estimate of the added sugars was performed based on the method proposed by the Pan American Health Organization Nutrient Profile Model, considering the quantity of total sugars declared (59). Even though this method estimates the quantity of free sugars in the product, all the free sugar corresponding to the added sugars in the products are used as a reference (cereal bars and sweet whole grain cookies). The FoPNL models were applied to the top-right corner of the labels, as suggested by the Chilean legislation that regulates FoPNL (18), and the percentage of the main panel area of the packages they occupied followed the model guidelines. The octagon and triangle models followed the proportions established in the Chilean legislation and application manual (18), and the labels with magnifying glasses followed the proportions in the Brazilian legislation (23).

### Experimental procedure

The research questionnaire consisted of four steps. In the first one, participants provided some personal information such as weight (kg), height (cm), whether they were responsible for the household grocery shopping, and the frequency of consumption of the evaluated products. Sociodemographic information such as gender, age, geographical region, socioeconomic status, and education was not requested, once the research company already had such data.

In the second step, the participants were introduced to the 12 food labels, one by one, following the Latin square design, and answered three questions:

(i) does this product contain any nutrient in a higher quantity than recommended for a healthy diet? With the following answer options: “No,” “Yes, sugar,” “Yes, saturated fat,” and “Yes, sodium.” The participant could choose more than one answer;
(ii) show how healthy you consider this product by choosing one of the options on a seven-point scale, ranging from 1—“not healthy” to 7—“very healthy”;
(iii) show your intention to purchase this product by choosing one of the options on a seven-point scale, ranging from 1—“I would definitely not buy it” to 7—“I would definitely buy it.”

The third step of the questionnaire was applied only to the groups that looked at FoPNL labels (octagon, magnifying glass, and triangle). The participants observed the respective FoPNL model and answered eight questions that assessed consumer opinions and perceptions regarding visibility, ease of understanding, and credibility of the information, among other aspects related to front-of-package labeling. Such questions were adapted from the study by Khandpur et al. (60) and answered according to a seven-point Likert scale, ranging from 1—“I strongly disagree” to 7—“I strongly agree.”

In the fourth and last step of the questionnaire, the participants were asked to answer the General Health Interest questionnaire, a subscale of the Health and Taste Attitude Scales (61), translated to Portuguese (62) and validated. This questionnaire consists of eight questions designed to assess consumer interest in healthy eating, which were answered according to a seven-point Likert scale, ranging from 1—“I strongly disagree” to 7—“I strongly agree” (62).

### Data analysis

All statistical analyses were performed using the *Statistical Package for the Social Sciences* (SPSS) software, versions 20.0 and 28.0, at a significance level of 5%. Differences in the frequency of participant sociodemographic characteristics were assessed using the chi-square test.

To evaluate the understanding of nutritional information, a binary variable was created for each label/product to identify the correct answers, using the answers to the question “Does this product contain any nutrient in a higher quantity than recommended for a healthy diet?” (“No;” “Yes, sugar;” “Yes, saturated fat;” “Yes, sodium”). Since the participant could choose more than one option, an answer to this question was considered correct only if the participant mentioned those nutrients that were excessive. Answers that considered the nutrients in excess, as well as others whose values were not above the ones recommended by the nutritional profile adopted (23), were not considered correct. The percentage of correct answers was calculated for each FoPNL condition. A generalized linear mixed model (GLM), through binary logistic regression, with random effects on the individuals was used to evaluate the influence of FoPNL conditions (control, octagon, magnifying glass, and triangle), nutrition claim condition (presence and absence), food category (cereal bar, cookie, and snack), and type of product (containing one or two excessive nutrients) and their two-way interactions in the probability of a correct understanding of the nutritional information. The model was adjusted for questions about interest in health in which significant differences between experimental groups were detected.

Healthfulness perception and purchase intention scores were evaluated using a mixed model analysis of variance (ANOVA). FoPNL, nutrition claims, food category, type of product, and their interactions were considered fixed effects, and participants were considered random effects. When interactions were significant, ANOVA was performed for each type of product separately. Sidak’s test was used for a *post hoc* comparison of the average values. The models included, as adjustment variables, those questions about interest in health in which significant differences between experimental groups were detected.

The average and standard deviation of agreement regarding the Likert scale were calculated separately for each question of the opinion and perception questionnaire and the interest in health one. ANOVA was used to assess the existence of significant differences between the conditions of FoPNL, and Tukey’s test was used for *post hoc* comparisons.

## Results

### Participants

The samples consisted of 51.7% women and 48.3% men, and the median age was 30 years, ranging from 18 to 73 years (Table 2). Significant differences were found between groups solely regarding the consumption frequency of cereal bars and snacks (Table 2).

**TABLE 2:** Sociodemographic characteristics of the study participants.

Characteristic	Participant percentage (%)				
	General (*n* = 720)	Control (*n* = 180)	Octagon (*n* = 180)	Triangle (*n* = 180)	Magnifying glass (*n* = 180)
**Age (median/P25–p75)**	30 (23–41)	30 (24–39)	31 (23–42)	30 (23–42)	31 (23–41)
18–24 years old	33.3	33.3^a^	33.3^a^	33.3^a^	33.3^a^
25–34 years old	30.0	30.0^a^	30.0^a^	30.0^a^	30.0^a^
35–44 years old	17.2	17.2^a^	17.2^a^	17.2^a^	17.2^a^
45–54 years old	12.2	12.2^a^	12.2^a^	12.0^a^	12.2^a^
55 years old or older	7.2	7.2^a^	7.2^a^	7.2^a^	7.2^a^
**Gender**					
Female	51.7	51.7^a^	51.7^a^	51.7^a^	51.7^a^
Male	48.3	48.3^a^	48.3^a^	48.3^a^	48.3^a^
**BMI (median/P25–P75)**	24.7 (21.9–27.8)	24.5 (21.5–28.0)	25.4 (21.8–28.2)	24.5 (22.0–27.2)	24.4 (21.9–27.7)
Underweight	4.2	3.9^a^	5.0^a^	4.4^a^	3.3^a^
Normal weight	48.2	49.4^a^	40.0^a^	51.1^a^	52.2^a^
Overweight	32.8	29.4^a^	40.6^a^	31.1^a^	30.0^a^
Class I obesity	10.0	10.6^a^	9.4^a^	8.9^a^	11.1^a^
Class II obesity	4.0	5.6^a^	3.9^a^	3.3^a^	3.3^a^
Class III obesity	0.8	1.1^a^	1.1^a^	1.1^a^	0.0^a^
**Region**					
Midwest	7.8	7.8^a^	7.8^a^	7.8^a^	7.8^a^
North	8.9	8.9^a^	8.9^a^	8.9^a^	8.9^a^
Northeast	27.2	27.2^a^	27.2^a^	27.2^a^	27.2^a^
South	13.9	13.9^a^	13.9^a^	13.9^a^	13.9^a^
Southeast	42.2	42.2^a^	42.2^a^	42.2^a^	42.2^a^
**Education**					
Incomplete middle school education	38.9	38.9^a^	38.9^a^	38.9^a^	38.9^a^
Complete middle school/Incomplete high school education	12.8	12.8^a^	12.8^a^	12.8^a^	12.8^a^
Complete high school/Incomplete higher education	31.1	31.1^a^	31.1^a^	31.1^a^	31.1^a^
Complete higher education	12.5	12.8^a^	14.4^a^	10.6^a^	12.2^a^
Post-graduation education	4.7	4.4^a^	2.8^a^	6.7^a^	5.0^a^
**Socioeconomic status**					
Up to 2 minimum wages	16.7	17.8^a^	14.4^a^	15.6^a^	18.9^a^
2–4 minimum wages	13.3	12.2^a^	15.6^a^	14.4^a^	11.1^a^
4–10 minimum wages	55.6	55.6^a^	55.6^a^	55.6^a^	55.6^a^
10–20 minimum wages	13.2	13.9^a^	12.2^a^	12.8^a^	13.9^a^
Over 20 minimum wages	1.2	0.6^a^	2.2^a^	1.7^a^	0.6^a^
**Are you responsible for the grocery shopping?**					
Yes	93.2	95.6^a^	97.8^a^	87.8^b^	91.7^a,b^
No	6.8	4.4^a^	2.2^a^	12.2^b^	8.3^a,b^
**Frequency of cereal bar consumption**					
Every day	10.4	9.4^a^	10.6^a^	8.9^a^	12.8^a^
2–3 times a week	30.3	41.1^a^	31.1^a,b^	23.3^b^	25.6^b^
Once a week	17.4	18.3^a^	17.2^a^	18.9^a^	15.0^a^
2–3 times a month	27.9	22.8^a^	25.6^a^	30.6^a^	32.8^a^
Never	14.0	8.3^a^	15.6^a,b^	18.3^b^	13.9^a,b^
**Frequency of whole grain cookies consumption**					
Every day	10.1	11.7^a^	13.3^a^	7.2^a^	8.3^a^
2–3 times a week	37.1	37.8^a^	34.4^a^	37.2^a^	38.9^a^
Once a week	20.8	22.2^a^	20.0^a^	20.6^a^	20.6^a^
2–3 times a month	19.2	17.8^a^	22.2^a^	18.3^a^	18.3^a^
Never	12.8	10.6^a^	10.0^a^	16.7^a^	13.9^a^
**Frequency of snack consumption**					
Every day	3.5	5.6^a^	4.4^a^	1.7^a^	2.2^a^
2–3 times a week	26.5	36.7^a^	23.3^b^	20.0^b^	26.1^a,b^
Once a week	30.0	26.1^a^	31.7^a^	30.0^a^	32.2^a^
2–3 times a month	28.3	21.7^a^	27.8^a,b^	35.6^b^	28.3^a,b^
Never	11.7	10.0^a^	12.8^a^	12.8^a^	11.1^a^

Different letters on the same line indicate a significant difference using the chi-square test (*p* < 0.050).

### Understanding of nutritional information

The understanding of nutritional information was significantly affected by FoPNL, food category, type of product, nutrition claims, and by “FoPNL × food category” and “Food category × type of product” interactions (Table 3).

**TABLE 3:** *F* values and *p*-values of fixed effects and interactions of the GLM performed to the understanding of nutritional information and the ANOVA models used for healthfulness perception and purchase intention.

Effect	df	Understanding of nutritional information	Healthfulness perception	Purchase intention
	
		*F* (*p*-value)	*F* (*p*-value)	*F* (*p*-value)
FoPNL	3	**205.66 (<0.001)**	**88.46 (<0.001)**	**70.00 (<0.001)**
Food category	2	**4.50 (0.011)**	**7.48 (0.001)**	**116.00 (<0.001)**
Type of product	1	**73.31 (<0.001)**	**327.85 (<0.001)**	**99.25 (<0.001)**
Nutrition claims	1	**24.90 (<0.001)**	**57.82 (<0.001)**	**31.21 (<0.001)**
FoPNL*food category	6	**3.61 (0.001)**	1.62 (0.138)	**3.79 (0.001)**
FoPNL*type of product	3	1.30 (0.271)	**15.92 (<0.001)**	**10.04 (<0.001)**
FoPNL*nutrition claims	3	0.50 (0.681)	0.40 (0.753)	0.71 (0.545)
Food category*type of product	2	**4.54 (0.011)**	2.93 (0.053)	**39.70 (<0.001)**
Food category*nutrition claim	2	0.07 (0.936)	0.99 (0.371)	**3.57 (0.028)**
Type of product*nutrition claims	1	0.70 (0.403)	2.02 (0.155)	0.08 (0.780)
FoPNL*food category*type of product	6	0.50 (0.806)	0.84 (0.536)	**2.66 (0.014)**
FoPNL*food category*nutrition claims	6	0.90 (0.495)	1.53 (0.165)	0.70 (0.648)
FoPNL*type of product*nutrition claims	3	0.14 (0.934)	2.05 (0.105)	0.41 (0.742)
Food category*Type of product*nutrition claims	2	0.45 (0.635)	0.45 (0.638)	0.24 (0.782)
FoPNL*food category*type of product*nutrition claims	6	0.70 (0.651)	0.48 (0.823)	0.16 (0.988)

**ID Variance**		**Estimate (standard error); *p*-value**		

		3.393 (0.254); *p* < 0.001	0.320612 (0.17628); *p* < 0.001	0.342764 (019059); *p* < 0.001

df, degree of freedom. Bold indicates statistically significant findings at p < 0.05 level. Models adjusted for the questions about interest in health with p < 0.05 and random effects on individuals.

The presence of nutritional claims reduced by 32% the chance of answering correctly in relation to the content of critical nutrients (Table 4). The inclusion of FoPNL increased the chance of correct answers in the three food categories (Table 4). For the cereal bar category, for instance, the chance of answering correctly was 63 times greater for the octagon group and 54 and 44 times greater for the triangle and magnifying glass, respectively, when compared to the control (Table 4).

**TABLE 4:** Odds ratios (IC95%) of the significant effects and interactions of the model in the understanding of nutritional information.

Effect		Understanding of nutritional information
		correct answers
**Nutrition claims**		
Absence		1
Presence		0.68 (0.58–0.78)**
**Food category × FoPNL**		
Cereal bar	Control	1
	Octagon	63.28 (39.79–100.63)**
	Triangle	54.19 (34.13–86.03)**
	Magnifying glass	44.15 (28.12–69.31)**
Cookie	Control	1
	Octagon	91.25 (55.52–149.97)**
	Triangle	88.16 (53.44–145.44)**
	Magnifying glass	93.21 (56.70–153.24)**
Snack	Control	1
	Octagon	89.53 (54.01–148.40)**
	Triangle	85.91 (51.60–143.02)**
	Magnifying glass	108.85 (65.25–181.57)**
**Food category × type of product**		
Cereal bar	1 Excessive nutrient	1
	2 excessive Nutrients	0.51 (0.41–0.64)**
Cookie	1 Excessive nutrient	1
	2 Excessive nutrients	0.79 (0.63–0.99)*
Snack	1 Excessive nutrient	1
	2 Excessive nutrients	0.51 (0.41–0.65)**
Akaike information criteria		46,342.696

Model adjusted for the questions about interest in health with *P* < 0.05 and random effects on individuals. Significance was evaluated in relation to the reference category (1.00) at *p < 0.05 and **p < 0.01.

Comparing the products with one and two critical nutrients in excess, it was possible to observe that the greater amount of critical nutrients (i.e., two nutrients) reduced the understanding of nutritional information by 49, 21, and 49% for the cereal bars, cookies, and snacks, respectively (Table 4).

### Healthfulness perception

Healthfulness perception of products was significantly affected by all the main effects included in the model (FoPNL, food category, type of product, and nutrition claims) and by the interaction “FoPNL × type of product.” The significance of this interaction suggested that the impact of FoPNL on healthfulness perception depends on the number of critical nutrients in the product (1 or 2 nutrients in excess) (Table 3).

Table 5 shows the average health scores for the evaluated products, considering the effects included in the model, and their interactions. It is possible to observe that cereal bars were perceived as least healthy than cookies and snacks and the presence of nutrition claims statistically increased health perceptions (Table 5).

**TABLE 5:** Average healthfulness perception scores by FoPNL (control, octagon, triangle, and magnifying glass), food category (cereal bar, cookie, and snack), type of product (containing 1 and 2 excessive nutrients), nutrition claims (absence and presence), and their interactions.

Effect	Healthfulness perception Mean (95% Confidence interval)	
**Food category**		
Cereal bar	1.96 (1.92–2.01)^b^	
Cookie	2.00 (1.96–2.05)^a^	
Snack	1.99 (1.95–2.03)^a^	
**Nutrition claims**		
Absence	1.95 (1.91–2.00)^a^	
Presence	2.02 (1.98–2.06)^b^	

**FoPNL × type of product**	**1 excessive nutrient**	**2 excessive nutrients**

Control	2.61 (2.52–2.07)^b^ ^A^	2.55 (2.47–2.64)^c^ ^B^
Octagon	1.82 (1.73–1.91)^a A^	1.64 (1.56–1.73)^a B^
Triangle	1.84 (1.75–1.93)^a A^	1.63 (1.54–1.71)^a B^
Magnifying glass	1.98 (1.89–2.07)^a A^	1.82 (1.73–1.90)^b B^

Different lowercase letters on the same column and uppercase letters on the same line indicate a significant difference using Sidak’s test (*p* < 0.050). Model adjusted for the questions about interest in health with *p* < 0.05 and random effects on individuals.

FoPNL reduced the healthfulness perception in the two types of the evaluated products (1 or 2 nutrients in excess). However, it is important to note that for products with only one nutrient in excess, the FoPNL models did not differ from each other, all being more effective than the control. In products with two critical nutrients, the octagon and triangle significantly reduced the perception of healthfulness in relation to the magnifying glass and all models were superior to the control. The least healthy products (two excessive nutrients) received lower health scores than the products with one critical nutrient in excess, across all experimental arms.

### Purchase intention

Purchase intention was significantly affected by all the main effects of the model and by the interactions “FoPNL*food category,” “FoPNL*type of product,” “Food category*type of product,” “Food category*nutrition claim,” and “FoPNL*food category*type of product” (Table 3).

In the presence of claims, purchase intention was higher in the cookies and snacks categories, but for cereal bars, the inclusion of this information did not change consumers’ intentions (Table 6). The effect of FoPNL varied according to food category and type of product, reducing consumers’ purchase intention. In the snacks category, no statistical difference was observed between the evaluated FoPNL models in either of the two types of products. This also happened in the most healthy version of the cookie. However, considering the least healthy version of the cookie, the purchase intentions of consumers who saw the octagon and the triangle were lower than the ones who saw the magnifying glass (Table 6).

**TABLE 6:** Average purchase intention scores by FoPNL (control, octagon, triangle, and magnifying glass), food category (cereal bar, cookie, and snack), type of product (containing 1 and 2 excessive nutrients), nutrition claims (absence and presence), and their interactions.

Effect	Purchase intention Mean (95% confidence interval)					
Food category × nutrition claims	Absence			Presence		
Cereal bar	2.09 (2.01–2.14)^a A^			2.12 (2.07–2.17)^a A^		
Cookie	2.03 (1.98–2.07)^b A^			2.08 (2.03–2.12)^b B^		
Snack	1.88 (1.83–1.93)^c A^			1.97 (1.92–2.02)^c B^		

**FoPNL × food category × type of product**	**Cereal Bar**		**Cookie**		**Snack**	
	**1 excessive nutrient**	**2 excessive nutrients**	**1 excessive nutrient**	**2 excessive nutrients**	**1 excessive nutrient**	**2 excessive nutrients**

Control	2.64 (2.55–2.74)^c A^	2.64 (2.54–2.74)^c A^	2.57 (2.47–2.67)^b A^	2.65 (2.55–2.75)^c b^	2.54 (2.44–2.63)^b A^	2.42 (2.33–2.52)^b b^
Octagon	1.94 (1.84–2.04)^a A^	1.81 (1.71–1.90)^a,b B^	1.84 (1.74–1.94)^a A^	1.77 (1.67–1.87)^a B^	1.82 (1.72–1.92)^a A^	1.65 (1.55–1.75)^a B^
Triangle	1.95 (1.85–2.05)^a,b A^	1.72 (1.63–1.82)^a B^	1.83 (1.73–1.93)^a A^	1.81 (1.72–1.91)^a A^	1.80 (1.70–1.90)^a A^	1.56 (1.47–1.66)^a B^
Magnifying glass	2.15 (2.05–2.24)^b A^	1.99 (1.90–2.09)^b B^	1.92 (1.82–2.02)^a A^	2.01 (1.91–2.11)^b B^	1.92 (1.82–2.02)^a A^	1.68 (1.58–1.78)^a B^

Different lowercase letters on the same column and uppercase letters on the same line indicate a significant difference using Sidak’s test (*p* < 0.050). Model adjusted for the questions about interest in health with *p* < 0.05 and random effects on individuals.

In the cereal bar category, while in the most healthy version of the product, the octagon symbol reduced the purchase intention in relation to the magnifying glass and control, and in the least healthy version, only the triangle symbol had this effect. It is worth noting that in all experimental arms, participants had different purchase intentions among the most and least healthy versions of the evaluated products, except for the cereal bars without FoPNL (Table 6).

### Consumer perceptions of front-of-package nutritional labeling models

The opinions and perceptions of participants regarding the evaluated FoPNL models are shown in Table 7. No significant difference between models was observed in any of the questions presented and, for all of them, the median answer was 7.0. Participant interest in health only varied between the experimental groups for three of the eight questions in the questionnaire (Table 8).

**TABLE 7:** Participant perception of the FoPNL models evaluated.

Questions	FoPNL		
	Octagon	Triangle	Magnifying glass
	
	Average (SD)	Average (SD)	Average (SD)
(1) This front-of-package labeling model called my attention.	6.54 (1.02)^a^	6.55 (1.04)^a^	6.45 (1.06)^a^
(2) This front-of-package labeling model is visible.	6.57 (1.03)^a^	6.54 (0.97)^a^	6.52 (0.95)^a^
(3) This front-of-package labeling model is easy to understand.	6.68 (0.75)^a^	6.73 (0.74)^a^	6.73 (0.61)^a^
(4) This front-of-package labeling model will help me quickly decide which products to buy.	6.58 (0.86)^a^	6.49 (1.07)^a^	6.51 (0.86)^a^
(5) This front-of-package labeling model will help me identify healthier products.	6.52 (1.04)^a^	6.56 (1.09)^a^	6.55 (0.80)^a^
(6) This front-of-package labeling model will help me decide whether I should buy a product.	6.41 (1.13)^a^	6.36 (1.12)^a^	6.44 (0.74)^a^
(7) I consider the information of this front-of-package labeling model credible and truthful.	6.58 (0.89)^a^	6.45 (1.06)^a^	6.49 (0.81)^a^
(8) This front-of-package labeling model will change my decision of which products to buy.	6.34 (1.17)^a^	6.20 (1.32)^a^	6.29 (0.99)^a^

Different letters on the same line indicate a significant difference using Tukey’s test (*p* < 0.050).

**TABLE 8:** Participant interest in health, evaluated through the General Health Interest Questionnaire, by experimental condition.

Questions	FoPNL			
	Control	Octagon	Triangle	Magnifying glass
	
	Average (SD)	Average (SD)	Average (SD)	Average (SD)
(1) I am very worried about how healthy foods are.	6.03 (1.06)^a^	6.06 (1.13)^a^	5.90 (1.28)^a,b^	5.68 (1.24)^b^
(2) I always follow a healthy and balanced diet.	5.21 (1.36)^b^	4.90 (1.52)^a,b^	4.67 (1.62)^a^	4.84 (1.45)^a,b^
(3) It is important for me that my diet is low in fat.	5.47 (1.55)^a^	5.17 (1.65)^a,b^	4.81 (1.76)^b^	4.87 (1.65)^b^
(4) It is important for me that my daily diet contain many vitamins and minerals.	6.28 (0.98)^a^	6.10 (1.24)^a,b^	5.97 (1.22)^b^	6.02 (1.03)^a,b^
(5) I eat what I like and I DO NOT worry about how healthy the food is.	3.13 (2.04)^a^	3.39 (1.96)^a^	3.26 (2.01)^a^	3.23 (1.72)^a^
(6) How healthy the food is has little impact on my choices.	3.26 (2.07)^a^	3.30 (2.04)^a^	3.51 (2.00)^a^	3.38 (1.87)^a^
(7) How healthy snacks are does not make any difference for me.	3.19 (1.99)^a^	3.09 (1.91)^a^	3.23 (1.97)^a^	3.18 (1.88)^a^
(8) I DO NOT avoid any food, even those that may elevate my cholesterol.	2.81 (1.98)^a^	3.25 (2.05)^a^	2.91 (1.84)^a^	2.89 (1.77)^a^

Different letters on the same line indicate a significant difference using the Tukey’s test (*p* < 0.050).

## Discussion

Front-of-package nutritional labeling will be implemented in Brazil in October 2022 and will become mandatory for the vast majority of food and beverage producers in October 2023. However, so far, there has been little research on how Brazilian consumers understand and perceive different FoPNL models, especially the magnifying glass model, as it will be implemented in the country, as it will be implemented in the country. In this context, the present work compared the magnifying glass model with two FoPNL models in the warning format, in terms of understanding nutritional information, healthfulness perception, and purchase intention of Brazilian consumers.

### Effect on the understanding of nutritional information

The results found in the present study are aligned with the literature, which shows that FoPNL indeed favors the understanding of nutritional information (12, 14, 51, 60, 63). The presence of FoPNL, in the three evaluated models, increased the correct answers regarding the presence of critical nutrients in excess in the three evaluated food categories. Bandeira et al. (51) indicated a similar result, in which the octagon, the triangle, the circle, and magnifying glass symbols increased the understanding of the nutritional content, compared to the control, in an equal way.

On the contrary, the presence of claims and the greater quantity of excessive critical nutrients (i.e., the presence of two excessive nutrients) reduced the percentage of correct answers, in the three evaluated food categories, indicating that such information hinders consumer understanding. Some studies have reported that the overload of information in food labels may discourage consumers from seeking more nutritional information on packages as a way to minimize cognitive effort (33, 64), jeopardizing choices and, consequently, the change in consumer behavior (65).

### Effect on healthfulness perception and purchase intention

As hypothesized, the presence of nutrition claims on the evaluated labels generated a positivity bias, as it increased the participants’ healthfulness perceptions of food and purchase intentions. The results from the literature, concerning the influence of claims in foods with FoPNL on healthfulness perception and purchase intention, are mixed (32, 33, 39, 40, 50, 66), and some studies have observed a positivity bias of claims, as reported in the present study (39, 40). Such influence seems to be related to the type of claim presented on labels (39, 40, 67). For example, Mediano Stoltze et al. (39) observed, using breakfast cereal packages, that fiber-related claims led to more positive product ratings compared to having no claims or having fat-related claims. Furthermore, according to Talati et al. (44), claims may be less persuasive for foods considered “unhealthy,” which justifies the observed effect in the present study, especially in relation to cereal bars, which received the lowest health scores and their purchase intentions were not affected by the inclusion of nutrition claims.

Although such claims refer to positive nutritional properties, they are frequently presented in products with unbalanced nutritional profiles (68–71). This is particularly worrisome, once, as in the present study, other studies have already shown that the presence of claims on food labels tends to increase their healthfulness perception (15, 39, 72–74). Thus, some countries, such as Australia, New Zealand (75), and Mexico (19), forbid claims on foods that contain excessive content of critical nutrients.

The inclusion of FoPNL statistically reduced healthfulness perceptions and purchase intentions of the evaluated foods. In products with two critical nutrients, the octagon and the triangle had a greater effect than the magnifying glass in reducing the perception of healthfulness. As for the outcome of purchase intention, although all FoPNL models were greater than the control, for the cereal bars category and for the least healthy version of cookies, there was a better performance of the octagon and the triangle symbols in relation to the magnifying glass. These findings can be explained by the number of seals applied to the labels in the different FoPNL conditions. In the condition of the warning symbols (octagon and triangle), for each nutrient in excess, one seal was applied to the label. While in the magnifying glass condition, regardless of the number of critical nutrients, only one magnifying glass symbol was present on food labels.

This result can be discussed under the aspect of processing fluency, which concerns the ease or difficulty of processing new information (76). This fluency can be influenced by a large number of variables, such as the “figure-ground contrast, the clarity with which a stimulus is presented, the duration of its presentation, or the amount of previous exposure to the stimulus,” and can be objectively measured, through processing speed and accuracy (76). According to Delivett et al. (77), easily processed images and texts provide feelings of processing fluency that can shape people’s judgment of information. Some studies have already demonstrated this effect, where an increase in processing fluency has led to more positive product reviews (77–79).

In the present study, probably, the presence of two warning seals vs. a single magnifying glass, contemplating the same nutritional information, improving the processing fluency, and reducing the healthfulness perceptions and purchase intention (in the cereal bars category and for the less healthy version of cookies) of the consumers. Deliza et al. (12) observed that participants needed less time to identify the high nutrient content in the presence of the black octagon and triangle, compared to the magnifying glass, which may also reflect greater processing fluency in the presence of the warning symbols, since this fluency can be measured by processing speed and accuracy (76). Those authors (12) attribute this finding to the fact that warning symbols are more familiar and this has also been discussed by other authors (51, 52). However, the results of the present study demonstrate that not only the FoPNL design but also the number of seals applied to the labels influence consumers’ perceptions. Furthermore, in the present study, FoPNL was applied according to the requirements of the legislation, so that the smallest labeling area for the magnifying glass could also contribute to the worst results of this symbol.

Hodgkins et al. (80) suggest that labels with a data approach (nutrient-specific) require greater cognitive effort from the consumer for understanding, while the criteria-based approach (synthesis) requires less cognitive effort from the consumer. In this way, the use of two warning symbols—as a visual synthesis of two nutrition warnings, could help the consumer to understand the message more than just one symbol with two warning texts. However, a specific study would need to be designed for this.

### Combined effect of front-of-package nutritional labeling and claims

In the present study, no interaction between nutrition claims and FoPNL was observed in the evaluated outcomes, contradicting hypothesis 2. Despite the positive effects of FoPNL, as the increase in understanding of nutritional information and the reduction of healthfulness perception and purchase intention, this information could not cancel the positivity bias generated by the claims, which seems to happen independently. Other studies have evaluated the relative impact of FoPNL information and claims on consumers, obtaining diverse results, mainly in relation to the effect of the claims. However, while some studies observe the occurrence of the positivity bias (32, 39, 67), others do not find it (33, 34). In general, the results indicate a stronger effect of FoPNL information on consumers, compared to the claims (32, 39–41, 50, 67, 81). It was also observed in the present study since the F values for FoPNL were greater than the ones for claims in all the evaluated outcomes. Mediano Stoltze et al. (39) tested the co-occurrence of warning labels and nutrient content claims on consumers’ perception of products and behavioral intentions using breakfast cereal labels. They found similar results to those obtained in the present study—although the claims generated a positivity bias, the FoPNL presented a stronger effect, with no interaction with the claims (39). On the contrary, in the study by Talati et al. (82), the authors concluded that FoPNLs are more effective in improving food choices by consumers when nutrition and health claims are not present. These differences seem to be justified both by factors that influence the effect of claims on consumers and by differences in experimental design, such as the evaluated categories and types of food. The similarity between the results of this study and those found by Mediano Stoltze et al. (39), for example, may be due to the similarities between foods (foods with a “healthy connotation”) and the nutrition claims tested in both studies.

Such findings help support regulations on FoPNL, providing evidence concerning the influence of claims on FoPNL. According to McLean et al. (41), nutrition claims are only useful when they are consistent with the nutritional profile of foods, which is hardly ever a reality (68–71). For instance, Duran et al. (69) evaluated a sample of 3,491 products available in the Brazilian market and observed that foods with nutrition claims were more likely to be high in critical nutrients when compared to those with low content of critical nutrients. The presence of claims in “unhealthy” foods has also been reported by other authors (71, 83).

### Consumer perceptions of the front-of-package nutritional labeling models

With regards to participants’ opinions and perceptions about the respective evaluated FoPNL models, the “high grades” found and the lack of significant difference in the results support the efficacy of FoPNL concerning visibility, ease of understanding, and credibility of the information assessed through the questionnaire. Nonetheless, it is noteworthy that the octagon and the triangle models, as applied in this study, indicated better results than the magnifying glass, and all showed better results compared to the control in relation to healthfulness perception and purchase intention. According to Khandpur et al. (60), who evaluated consumers’ opinions about the triangle and the traffic light FoPNL models, although those opinions are important, label appeal may not mean that consumers will use labels in a better way. Likewise, their opinion about a piece of information may have a different impact on their behavior or behavior intention, which corroborates with the findings of the present study.

### Strengths and limitations

It is worth mentioning that this research is the first to evaluate the influence of nutrition claims on FoPNL, including the magnifying glass model, in the Brazilian population. This study was also carefully designed by applying the FoPNL dimensions as defined in the Brazilian legislation for the magnifying glass model (23) and in the Chilean legislation for the octagon and triangle models (18), collecting results closer to reality. Besides, the sample was meticulously composed of age-diverse participants and represented the Brazilian population regarding gender, socioeconomic status, education, and country region.

However, the present research has some limitations. The experimental design performed online may not represent the reality of purchases in physical stores and actual product labels. Nonetheless, it is believed that this limitation was minimized by excluding mobile devices (cell phones and tablets) from the study, allowing the visualization of food labels and their constituents in dimensions close to real ones on computer/laptop screens. In addition, online shopping has increased considerably, which requires understanding how consumers behave in this environment. Another aspect to be highlighted refers to the nutrition claims on the labels. For the three models, they were applied near the center of the front panel of the packages to promote similarity with the way claims appear on actual products. Thus, their application did not follow the parameter established by the new Brazilian legislation, which states that nutrition claims should be placed on the lower half of the frontal panel, with smaller characters than those used in the magnifying glass FoPNL (24). Moreover, the effect of the different types of nutrition claims that were included on labels was not evaluated, to assess possible differences in participants’ associations.

The question about participants’ weight before the application of the questionnaire and the order in which the outcomes were evaluated in the questionnaire may have triggered a response bias. The question about nutrient content, presented first, may have influenced the perception of healthfulness and this, in turn, influenced the purchase intention. Another issue that should be mentioned as a limitation is that changing both the design and the size of the FoPNL, based on the application criteria of Brazilian and Chilean legislation, makes it difficult to conclude which factor was responsible for the differences found in results. It is also worth noting that the results may not be generalizable to a larger variety of foods, once only three food categories were evaluated.

## Conclusion

In general, the presence of FoPNL favored the understanding of nutritional information and reduced healthfulness perception and purchase intention. On the contrary, nutrition claims reduced the percentage of correct answers regarding excessive nutrients and increased healthfulness perception and purchase intention. Despite the positive effects of FoPNL, it did not cancel the positivity bias generated by the claims, which occurred independently. However, the effect of FoPNL varied according to the food category and the amount of critical nutrients in the product, with those with two nutrients in excess receiving the lowest health and purchase intentions scores.

Regarding the FoPNL models, all were more effective than the control. However, for the least healthy type of product (2 nutrients in excess), the octagon and the triangle models were superior to the magnifying glass, regarding the outcome of healthfulness perception. Finally, we consider that the results of this study constitute evidence that proves and reinforces the efficacy and benefits of including FoPNL for consumer understanding and judgment of the healthfulness perception and purchase intention of products. Future research that evaluates FoPNL effectiveness on consumer behavior changes, as well as research that evaluates the effect of different types of claims on consumers’ associations and specific research on the understanding of the symbols and attention to them, may be interesting to complement the body of evidence regarding FoPNL inclusion on food labels.

## Data availability statement

The original contributions presented in this study are included in the article, further inquiries can be directed to the corresponding author/s.

## Ethics statement

The studies involving human participants were reviewed and approved by Research Ethics Committee of the Universidade Federal de Minas Gerais (Brazil Platform–CAAE 2395020.1.0000.5149). The participants provided their written informed consent to participate in this study.

## Author contributions

SP and LA: study conception and manuscript. CR and CS: label creation. SP, LA, and IR: statistical analysis. SP, IR, LA, and CR: data analysis and discussion. All authors reviewed and approved the final version submitted.
